# Indigenous Soil Microbiota Shapes *Bacillus subtilis*-Mediated Biocontrol Against Potato Bacterial Wilt Revealed by Integrated Rhizosphere Microbiome and Metabolome Profiles

**DOI:** 10.3390/plants15213252

**Published:** 2026-10-22

**Authors:** Feng Zhang, Xianjun Lai, Zhouhua He, Shuyan Wang, Shifeng Liu, Xiyao Wang, Lang Yan

**Affiliations:** 1 Potato Research and Development Center, College of Agronomy, Sichuan Agricultural University, Chengdu 611130, China; zhangfeng_95@126.com (F.Z.); 17516302992@163.com (Z.H.); wwssyy0913@163.com (S.W.); 2 Panxi Crop Improvement Key Laboratory of Sichuan Province, College of Agriculture Science, Xichang University, Liangshan 615000, China; laixianj@hotmail.com (X.L.); 13838657723@163.com (S.L.)

**Keywords:** *Bacillus subtilis*, biocontrol, indigenous soil microbiota, metabolomics, potato bacterial wilt, rhizosphere microbiome

## Abstract

The inconsistent field performance of microbial biocontrol agents is often linked to resident soil microbiota, yet how indigenous microbiota influence *Bacillus*-mediated suppression of potato bacterial wilt remains insufficiently understood. In this study, sterilized and non-sterilized soils were used to evaluate how indigenous soil microbiota affect the biocontrol efficacy of *Bacillus subtilis* against *Ralstonia solanacearum*. Disease development, pathogen abundance, *B. subtilis* abundance, full-length 16S rRNA amplicon sequencing, and untargeted metabolomics were integrated to characterize rhizosphere microbial and metabolic responses. Soil microbial background strongly influenced disease progression and *Bacillus* performance. Under sterilized-soil conditions, *R. solanacearum* abundance remained 3–5-fold higher and disease severity increased more rapidly, whereas in non-sterilized soil, *B. subtilis* significantly reduced disease index and pathogen abundance. Microbiome analyses showed that sterilized soil was associated with limited *Bacillus*-mediated pathogen buffering, whereas non-sterilized soil supported the enrichment of potentially beneficial taxa, including *Burkholderia*, *Devosia*, and *Rhizobium*, together with soil-context-dependent microbial association patterns and shifts in predicted functional potential. Metabolomic profiling further revealed that *Bacillus*-associated metabolic profiles were highly soil-context-dependent, with low overlap between sterilized and non-sterilized soils under *Bacillus* treatment, as indicated by a Jaccard similarity of 0.059. In non-sterilized soil, *Bacillus* treatment was associated with defense-related metabolic profiles involving aromatic defense-related compounds and metabolites associated with antimicrobial activity and systemic resistance, whereas sterilized soil showed stronger damage-compensation-associated metabolic patterns. These findings suggest that indigenous soil microbiota may shape the ecological expression of *B. subtilis*-mediated biocontrol and highlight the importance of considering resident microbial backgrounds for potato bacterial wilt biocontrol.

## 1. Introduction

Potato bacterial wilt, caused by *Ralstonia solanacearum*, is one of the most devastating soil-borne diseases affecting solanaceous crops worldwide, leading to severe yield losses and posing a persistent threat to sustainable agriculture [1,2]. Due to its wide host range, high genetic diversity, and strong environmental adaptability, effective management of this pathogen remains challenging [3,4]. Conventional control strategies often provide limited and unstable protection, particularly under field conditions where complex soil environments influence disease development [5]. As a result, increasing attention has been directed toward biological control using beneficial microbes, especially *Bacillus* species, as a promising and environmentally friendly approach to suppress bacterial wilt [6,7]. Numerous studies have demonstrated that *Bacillus* strains can inhibit *R. solanacearum* through multiple mechanisms, including the production of antimicrobial compounds, competition for ecological niches, and induction of systemic resistance in host plants [8,9]. For example, *Bacillus*-based formulations have been reported to significantly reduce disease incidence in tomato and potato by enhancing plant defense responses and suppressing pathogen proliferation in the rhizosphere [10]. However, despite these advances, the efficacy of *Bacillus*-mediated biocontrol remains highly variable across different soils and environments, often showing promising results under controlled conditions but inconsistent performance in the field. This discrepancy suggests that *Bacillus* efficacy is not solely determined by strain properties but may strongly depend on the ecological context of the resident soil microbiome.

Increasing evidence suggests that indigenous soil microbiota may be a major factor shaping why the same biocontrol agent performs effectively in some soils but inconsistently or fails in others [11]. The rhizosphere microbiome is a complex and dynamic ecological network, where microbial interactions, rather than individual species, regulate pathogen invasion and plant health [12]. Previous studies have shown that disease-suppressive soils harbor specific microbial consortia that can inhibit pathogens through cooperative interactions. In contrast, disruption of the native microbiota, such as through soil sterilization, is often associated with increased disease susceptibility and unstable microbial assembly [13,14]. Nevertheless, most existing studies have primarily examined pathogen suppression or microbiome composition separately, while the tripartite interaction among indigenous microbiota, pathogen invasion, and introduced *Bacillus* remains poorly resolved. In particular, it remains unclear whether *Bacillus* functions mainly as an autonomous antagonist or whether its field efficacy fundamentally depends on indigenous microbiota-mediated functional amplification. Moreover, the mechanisms by which soil microbial background influences the assembly of rhizosphere communities and their associated metabolic profiles during disease progression have not been fully elucidated. Addressing these questions requires an experimental system that can compare the effects of introduced biocontrol agents under contrasting native microbiome backgrounds.

To investigate factors shaping *Bacillus* biocontrol success across soil environments, we employed a controlled comparison between sterilized and non-sterilized soil systems to evaluate the role of the indigenous microbiome in *Bacillus*-mediated suppression of potato bacterial wilt. We hypothesized that indigenous soil microbiota would strongly influence the efficacy and durability of *Bacillus*-mediated biocontrol by modulating pathogen abundance, rhizosphere microbial community structure, predicted functional potential, and defense-related metabolic profiles. By integrating full-length 16S rRNA gene sequencing with untargeted metabolomics, we systematically analyzed the dynamic changes in microbial community structure and metabolic profiles at different stages of disease progression. This design allowed us to (i) evaluate how indigenous microbiota shapes *Bacillus* functional expression under pathogen stress, (ii) quantify how soil microbial background alters *Bacillus* efficacy and durability, and (iii) explore microbiome–metabolome association patterns underlying soil-dependent biocontrol outcomes. This study supports indigenous soil microbiota as a critical ecological factor shaping *Bacillus* biocontrol performance and provides a conceptual basis for developing microbiome-informed biocontrol strategies.

## 2. Results

### 2.1. Effects of Soil Microbial Background on Disease Progression and Bacillus Biocontrol Efficacy

**Figure 1:**
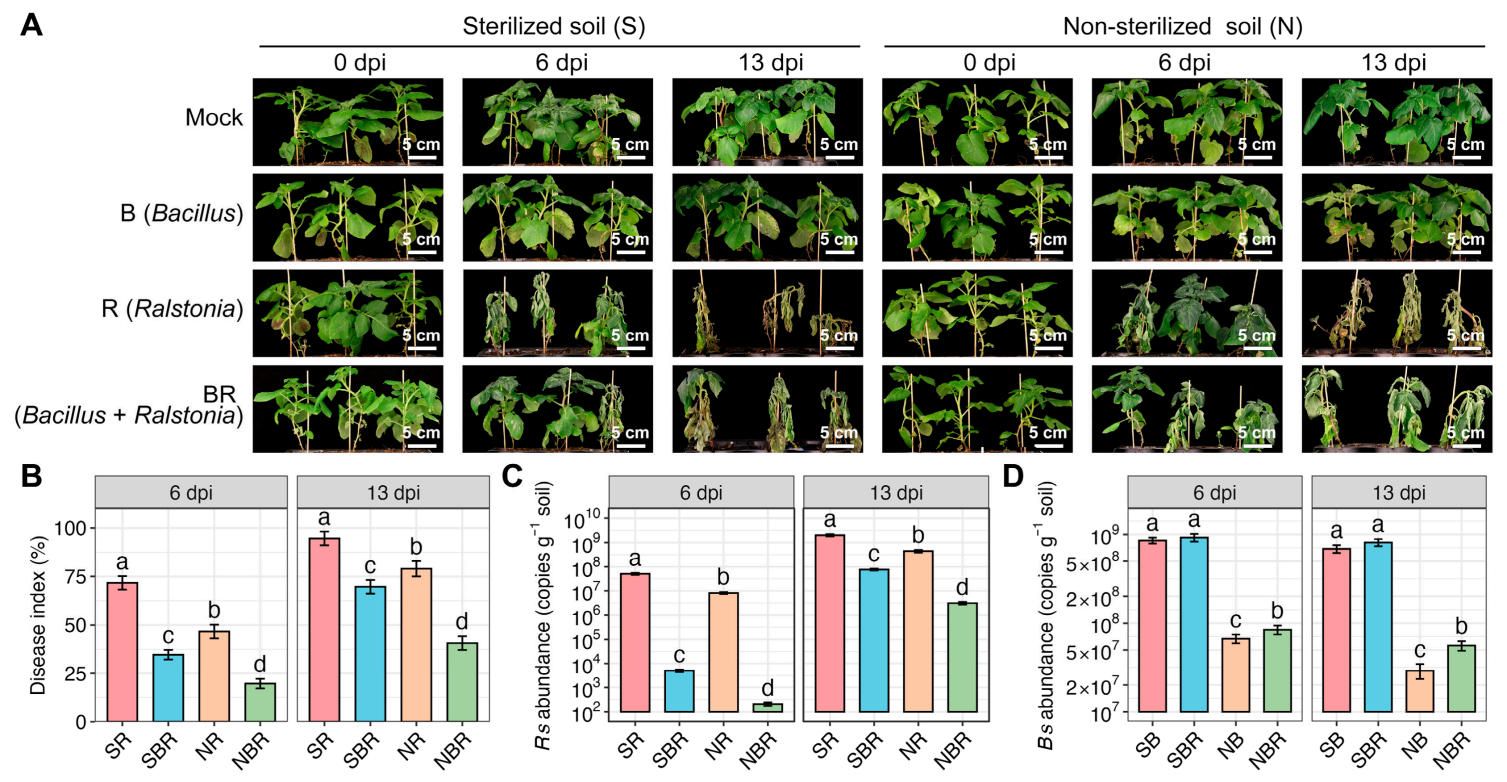
Effects of soil microbial background on potato bacterial wilt progression and *Bacillus*-mediated biocontrol under pathogen stress. (**A**) Representative phenotypes of potato plants grown in sterilized soil (S) and non-sterilized soil (N) under Mock, *Bacillus* (B), *Ralstonia* (R), and *Bacillus*+*Ralstonia* (BR) treatments at 0, 6, and 13 days post-inoculation (dpi). (**B**) Disease index (%) of pathogen-inoculated plants with or without *Bacillus* treatment under sterilized and non-sterilized soil conditions at 6 and 13 dpi. (**C**) Quantitative abundance of *Ralstonia solanacearum* in rhizosphere soil (copies g^−1^ soil) under corresponding treatments at 6 and 13 dpi, determined by qPCR. (**D**) qPCR quantification of *Bacillus subtilis* abundance in rhizosphere soil under *Bacillus*-treated conditions at 6 and 13 dpi. Data are shown as means ± SD of three independent pots. Each pot contained three plants, and the pot-level mean was used as one biological replicate. Different lowercase letters indicate significant differences among treatments within the same sampling time point based on two-way ANOVA followed by Tukey’s HSD test (*p* < 0.05). For disease index and *R. solanacearum* abundance, comparisons were performed among pathogen-inoculated groups; for *B. subtilis* abundance, comparisons were performed among *Bacillus*-treated groups.

To evaluate how indigenous soil microbiota influences disease development and *Bacillus*-mediated biocontrol, potato plants were grown in non-sterilized (N) and sterilized (S) soils under control (CK), *Bacillus* inoculation (B), pathogen inoculation (R), and *Bacillus* plus pathogen (BR). Disease progression was evaluated at the pot level using three independent pots per treatment, with three plants grown in each pot, and monitored at 6 and 13 dpi (days post-inoculation). Soil microbial background profoundly influenced both disease trajectory and biocontrol performance (Figure 1).

Under pathogen inoculation, disease symptoms developed earlier and more severely in sterilized soil (Figure 1A). At 6 dpi, SR plants already exhibited faster wilting progression than NR plants, and by 13 dpi this difference became substantially greater, with SR showing markedly stronger disease severity. These results indicated that sterilized soil conditions, characterized by disruption of indigenous microbiota and potential changes in soil abiotic properties, were associated with reduced rhizosphere buffering capacity and accelerated pathogen-driven disease progression. *Bacillus* alone caused no visible adverse effects on plant growth.

*Bacillus* reduced disease severity under both soil conditions, but its efficacy was strongly soil-background-dependent. In non-sterilized soil, NBR showed substantially lower symptom severity and disease index than NR, whereas in sterilized soil, although SBR partially alleviated disease relative to SR, plants still displayed pronounced wilting (Figure 1B). This suggested that while *Bacillus* possessed direct biocontrol potential, its stronger and more durable protective efficacy was associated with the presence of indigenous microbial support.

Pathogen quantification strongly paralleled disease severity (Figure 1C). Following pathogen inoculation, *R. solanacearum* abundance remained consistently 3–5-fold higher in sterilized soil than in non-sterilized soil at both stages. *Bacillus* reduced pathogen abundance by up to three orders of magnitude at 6 dpi in both soils. However, by 13 dpi, pathogen suppression durability diverged sharply between microbial backgrounds. In non-sterilized soil, BR maintained approximately 100-fold pathogen suppression, whereas in sterilized soil suppression declined to ~25-fold with stronger pathogen rebound. These findings indicated that the non-sterilized soil microbial background may restrict early pathogen expansion and contribute to the durability of *Bacillus*-mediated pathogen suppression.

To further determine whether the stronger biocontrol effect in non-sterilized soil was simply explained by higher abundance or relative persistence of the introduced *Bacillus*, we quantified *B. subtilis* abundance in the rhizosphere by qPCR (Figure 1D). *B. subtilis* signals were clearly detected in *Bacillus*-treated rhizosphere samples at both 6 and 13 dpi, whereas background signals in Mock and R-only treatments were negligible. No detectable *gyrB* amplification was observed in the non-inoculated sterilized-soil controls (SCK and SR), whereas low background signals (1.0–3.0 × 10^4^ copies g^−1^ soil) were detected in the non-sterilized controls (NCK and NR), approximately three to four orders of magnitude lower than those in *B. subtilis*-inoculated treatments. Among the *B. subtilis*-inoculated treatments, gyrB gene copy numbers were consistently higher in sterilized soil than in non-sterilized soil, regardless of pathogen inoculation. However, despite lower *B. subtilis* abundance in non-sterilized soil, NBR exhibited stronger and more durable suppression of pathogen than SBR. These results suggested that the enhanced biocontrol efficacy observed in non-sterilized soil was not simply attributable to higher *Bacillus* abundance but might be associated with the indigenous microbial background.

### 2.2. Soil Microbial Background Shapes Rhizosphere Microbiome Disruption and Bacillus-Associated Community Shifts Under Pathogen Stress

**Figure 2:**
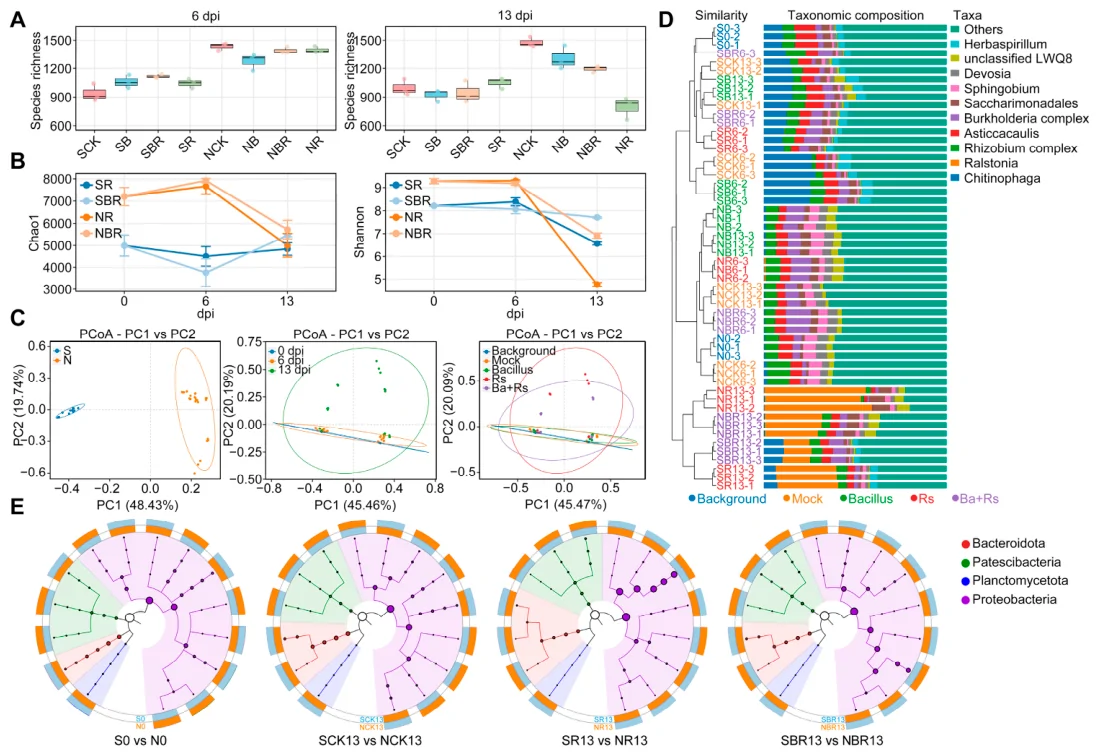
Soil microbial background shapes rhizosphere bacterial diversity, community structure, and phylogenetic shifts under pathogen stress and *Bacillus* intervention. (**A**) Species richness of rhizosphere bacterial communities under sterilized (S) and non-sterilized (N) soil backgrounds across treatments at 6 and 13 dpi. (**B**) Temporal dynamics of Chao1 richness and Shannon diversity indices across major treatment groups from 0 to 13 dpi. (**C**) Principal coordinate analysis (PCoA) of Bray–Curtis distances showing beta diversity patterns among samples at 0, 6, and 13 dpi. (**D**) Hierarchical clustering and relative taxonomic composition of rhizosphere bacterial communities across all samples based on species-level abundance profiles. (**E**) GraPhlAn phylogenetic cladograms showing major phylogenetic composition and taxonomic shifts among representative treatment comparisons under different soil microbial backgrounds. Data in diversity analyses are presented as mean ± SD. Treatments include Mock, *Bacillus* (B), *Ralstonia* (R), and *Bacillus*+*Ralstonia* (BR).

High-quality PacBio sequencing generated 3,296,324 CCS reads from 54 rhizosphere samples, yielding 15,893 ASVs and 3287 annotated species, providing sufficient resolution for microbial community analysis. Overall, non-sterilized soil consistently maintained greater species richness and broader microbial diversity than sterilized soil, whereas sterilized soil exhibited a lower-diversity microbiome structure. (Figure 2A,B). At 13 dpi, pathogen inoculation substantially reduced richness and alpha diversity in non-sterilized soil, with NR showing the strongest decline. *Bacillus* partially alleviated this decline in non-sterilized soil, as NBR maintained significantly higher diversity than NR, although not fully returning to healthy levels. In contrast, sterilized soil exhibited uniformly lower diversity and comparatively smaller treatment-induced fluctuations.

Beta diversity analysis (Figure 2C) further demonstrated that soil microbial background was a major factor associated with rhizosphere community structure, exerting a stronger influence than either pathogen or *Bacillus* treatment alone. Sterilized and non-sterilized soils were clearly separated along PC1, indicating that indigenous microbiota strongly contributed to the baseline rhizosphere community framework. Across disease progression, microbial communities shifted from tightly clustered baseline states at 0 dpi toward increasingly dispersed configurations at 13 dpi, with pathogen-treated samples showing the strongest deviation. Replicate dispersion was generally lower in baseline and CK groups but increased under pathogen-stressed treatments, particularly at 13 dpi, indicating stronger rhizosphere community heterogeneity during disease progression. *Ralstonia* samples exhibited the greatest dispersion and structural displacement, indicating that pathogen invasion was the primary driver of microbiome dysbiosis. *Bacillus* treatment substantially mitigated this shift, positioning BR communities between pathogen-only and healthy groups, suggesting that *Bacillus* primarily functions by buffering pathogen-associated community displacement rather than strongly restructuring healthy communities. Importantly, the PCoA included CK and *Bacillus*-only treatments at the corresponding stages, providing a baseline evaluation of *Bacillus* effects in the absence of pathogen pressure. Consistently, *Bacillus*-only treatments clustered closely with controls, suggesting that *Bacillus* alone had limited effects on native microbiome structure under these conditions.

Taxonomic composition (Figure 2D, Supplementary Figure S1) reinforced these community-level patterns by showing that sterilized soils were dominated by relatively few taxa, particularly *Chitinophaga silvisoli* and *Asticcacaulis taihuensis*, reflecting reduced bacterial complexity, whereas non-sterilized soils harbored more diverse indigenous taxa including *Sphingobium*, *Burkholderia*, and *Rhizobium*, highlighting the role of native microbiota in sustaining community complexity. At the phylogenetic level, GraPhlAn phylogenetic analysis (Figure 2E) extended this observation by revealing that although baseline major phylum composition remained relatively stable across soil backgrounds, pathogen stress consistently shifted rhizosphere communities toward Proteobacteria enrichment and Patescibacteria reduction. This shift became more consistent following *Bacillus* treatment (Supplementary Figure S2), indicating that *Bacillus* was associated with directional shifts in pathogen-disrupted rhizosphere communities.

### 2.3. Bacillus Suppresses Pathogen-Associated Dominance with Shifts in Microbial Biomarkers, Predicted Functions, and Association Networks

**Figure 3:**
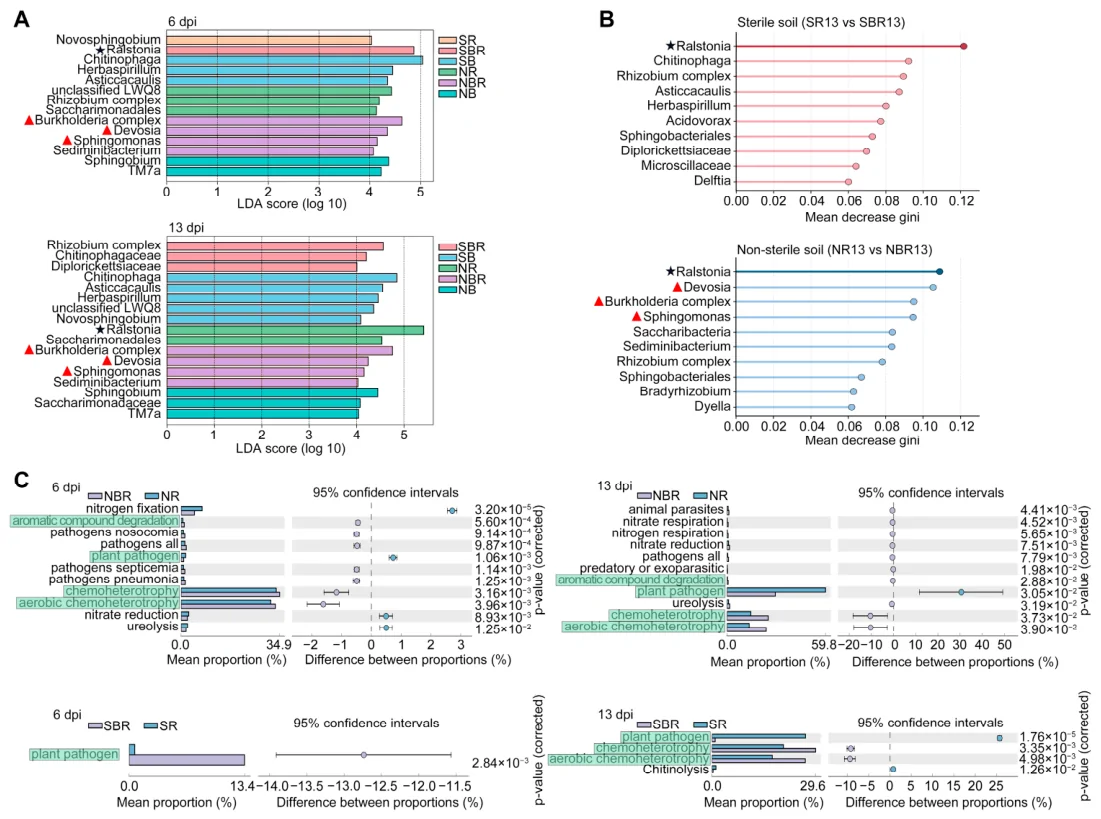
Bacillus is associated with shifts in rhizosphere microbial biomarkers, predicted functions, and co-occurrence networks under pathogen stress in a soil-background-dependent manner. (**A**) LEfSe analysis showing differentially enriched microbial taxa among treatment groups at 6 and 13 dpi under sterilized and non-sterilized soil conditions. Black stars highlight *Ralstonia*, whereas red triangles indicate representative potentially beneficial taxa. (**B**) Random forest analysis identifying major microbial biomarkers distinguishing Bacillus-treated and pathogen-inoculated communities under sterilized (SR13 vs. SBR13) and non-sterilized (NR13 vs. NBR13) soil conditions. The same symbols are used to highlight the corresponding taxa. (**C**) FAPROTAX-based prediction of potential microbial functional categories. Predicted functional categories related to pathogen-associated profiles and carbon/aromatic compound metabolism are highlighted. Predicted functions do not represent directly measured gene expression or metabolic activity.

Based on the baseline patterns observed in Figure 2C, subsequent biomarker, predicted functional, and network analyses focused on pathogen-stressed treatments, namely SR, SBR, NR, and NBR, to specifically characterize soil-context-dependent *Bacillus*-associated responses under disease pressure. LEfSe analysis showed that pathogen invasion and *Bacillus* intervention drove directional microbiome divergence as early as 6 dpi, and these differences became further consolidated into distinct ecological states by 13 dpi (Figure 3A). Under pathogen treatment, *Ralstonia*-associated taxa progressed from early enrichment to late-stage dominance, particularly in NR where *Ralstonia* became the strongest discriminatory biomarker, reflecting sustained pathogen expansion and pathogen-associated community shift. In contrast, *Bacillus*-treated NBR communities showed early enrichment of taxa, including *Burkholderia*, *Devosia*, and *Sphingomonas*, which remained relatively enriched through late stages. In sterilized soil, microbiome responses were instead dominated by opportunistic taxa such as *Chitinophaga*, *Asticcacaulis*, and *Herbaspirillum*, suggesting more limited alternative functional profiles under a depleted indigenous microbial background. Random forest analysis further supported this soil-background-dependent divergence (Figure 3B). Although *Ralstonia* remained the primary predictor distinguishing pathogen-inoculated and *Bacillus*-treated groups across both soils, the surrounding discriminatory taxa differed substantially. In sterilized soil, classification was driven mainly by pathogen-associated and opportunistic taxa, whereas in non-sterilized soil, genera including *Devosia*, *Burkholderia*, *Sphingomonas*, and *Sediminibacterium*, also emerged as major predictors, indicating that indigenous microbiota were associated with a broader range of discriminatory taxa following Bacillus treatment.

FAPROTAX-based functional prediction suggested that pathogen infection was associated with higher predicted plant pathogen-related functional potential, whereas *Bacillus*-treated communities showed higher predicted chemoheterotrophic and aromatic compound degradation potential (Figure 3C). In non-sterilized soil, NBR showed higher predicted functional potentials associated with chemoheterotrophy, aerobic chemoheterotrophy, and aromatic compound degradation, and these predicted profiles remained evident through 13 dpi, whereas NR remained strongly associated with predicted pathogen-related functional potential. In sterilized soil, SBR showed only partial shifts in predicted functional potential and remained more closely associated with predicted pathogen-related profiles, further suggesting that *Bacillus*-associated shifts in predicted functional potential differed between soil microbial backgrounds.

**Figure 4:**
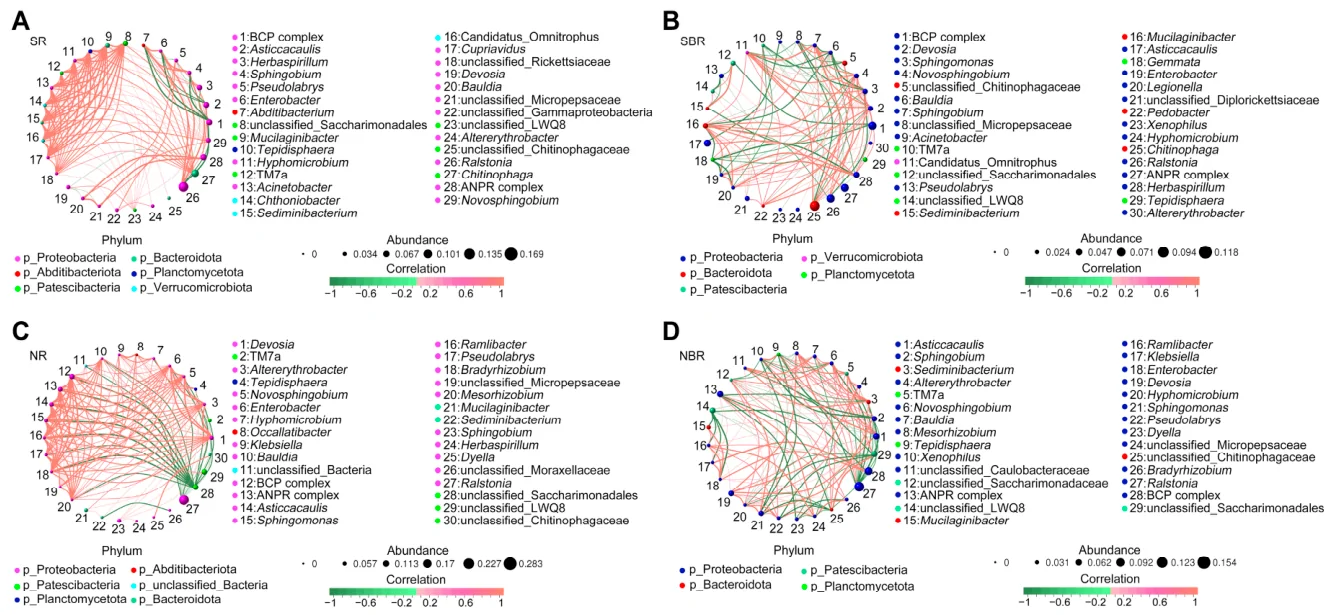
Rhizosphere bacterial co-occurrence networks under pathogen stress at 13 dpi. (**A**) SR13; (**B**) SBR13; (**C**) NR13; (**D**) NBR13. Full networks were constructed using genus-level relative abundance data by retaining all significant Spearman correlations with |ρ| > 0.8 and FDR-adjusted *p* < 0.05. Network topological properties were calculated from the full networks and are provided in Supplementary Table S1. For readability, the main figure shows the top 30 most abundant genera from each full network. Node size indicates relative abundance, node color indicates phylum, and edge color indicates positive or negative correlations. These networks represent correlation-based association patterns rather than direct ecological interactions. BCP complex, Burkholderia–Caballeronia–Paraburkholderia complex; ANPR complex, Allorhizobium–Neorhizobium–Pararhizobium–Rhizobium complex.

Co-occurrence network analysis provided an additional systems-level view of rhizosphere bacterial association patterns at 13 dpi (Figure 4, Supplementary Table S1). To avoid bias introduced by retaining an arbitrary fixed number of edges, full networks were reconstructed using a uniform threshold of Spearman’s |ρ| > 0.8 and FDR-adjusted *p* < 0.05. Network topological properties were calculated from the full threshold-based networks, whereas Figure 4 visualizes simplified networks containing the top 30 most abundant genera extracted from each full network for readability.

The full threshold-based networks revealed clear soil-background-dependent differences in network organization. In sterilized soil, SR13 contained 94 nodes and 1158 edges, whereas SBR13 contained 104 nodes and 1121 edges. Compared with SR13, SBR13 showed lower graph density, clustering coefficient, and betweenness centralization, suggesting a less tightly clustered and less centralized association structure after *Bacillus* treatment under pathogen stress. In non-sterilized soil, NBR13 showed more edges and a higher average degree than NR13, increasing from 975 to 1122 edges and from 23.78 to 25.21 in average degree, respectively. Meanwhile, graph diameter and betweenness centralization decreased from 15.72 to 11.89 and from 0.078 to 0.032, respectively, suggesting a more connected but less centralized association pattern in NBR13. *Ralstonia*-associated features further showed that pathogen-linked association patterns differed between soil backgrounds and were modified by *Bacillus* treatment. In sterilized soil, the number of positive correlations directly connected to *Ralstonia* decreased from 13 in SR13 to 6 in SBR13, whereas negative correlations increased slightly from 11 to 13. In non-sterilized soil, NR13 showed 32 direct negative correlations with *Ralstonia* and no positive correlations, whereas NBR13 showed 29 direct *Ralstonia*-associated correlations, including 7 positive and 22 negative correlations. Consistent with these full-network results, the top 30-node networks in Figure 4 showed that *Ralstonia* remained a large-abundance node in pathogen-inoculated treatments but was embedded in distinct soil-dependent association structures. In particular, non-sterilized networks contained several potentially beneficial or functionally relevant taxa, including *Devosia*, Burkholderia/Caballeronia/Paraburkholderia, and Rhizobium-related groups, whereas sterilized networks were more strongly represented by taxa such as *Chitinophaga*, *Asticcacaulis*, *Herbaspirillum*, and *Sphingobium*. Together, these results indicate that *Bacillus* treatment was associated with soil-background-dependent changes in the structure and taxonomic composition of *Ralstonia*-linked microbial associations, rather than a uniform increase in negative correlations. These networks represent correlation-based association patterns and should not be interpreted as direct ecological interactions.

### 2.4. Bacillus-Associated Metabolic Profiles Under Pathogen Stress Depends on Soil Microbial Background

**Figure 5:**
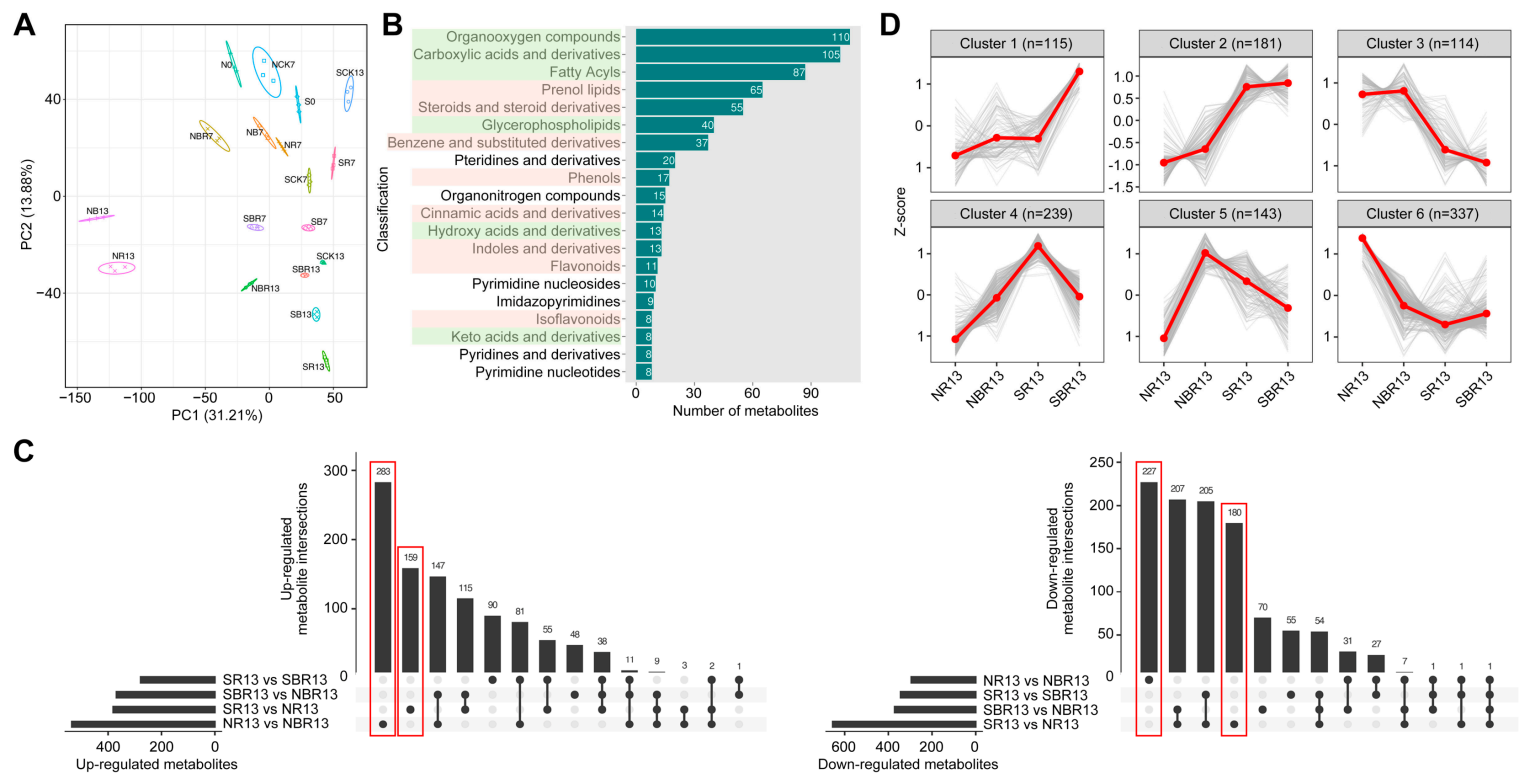
Global metabolomic profiling and differential metabolite patterns of rhizosphere soils under different soil microbial backgrounds and treatments. (**A**) Principal component analysis (PCA) of rhizosphere metabolomic profiles across all treatment groups and sampling stages. (**B**) Chemical classification and distribution of annotated metabolites detected in rhizosphere samples. Major basal metabolic classes and defense-/secondary-metabolism-related classes are highlighted in green and orange, respectively. (**C**) UpSet plots showing intersections of significantly upregulated and downregulated differential metabolites among major pairwise comparisons at 13 dpi. Red boxes highlight soil-context-specific *Bacillus*-associated metabolic responses in non-sterilized soil (NR13 vs. NBR13) and sterilized soil (SR13 vs. SBR13). (**D**) K-means clustering of differential metabolites across NR13, NBR13, SR13, and SBR13 groups, showing major dynamic metabolite clusters based on normalized abundance patterns. Metabolite clusters are presented as Z-score normalized abundance trajectories across treatment groups. Gray lines represent individual metabolite profiles, whereas red lines indicate the mean abundance trajectory of each cluster.

Untargeted LC-QTOF metabolomics annotated 1512 known metabolites across all samples. Quality control analyses showed high reproducibility, with 92.88% of detected metabolic features exhibiting QC RSD values ≤ 30% (Supplementary Figure S3). PCA revealed clear stage-dependent metabolic differentiation, with non-sterilized 13 dpi samples showing the greatest dispersion, indicating that under indigenous microbial conditions, late-stage pathogen stress combined with *Bacillus* intervention was associated with greater metabolic heterogeneity (Figure 5A). Although three biological replicates were used per treatment, replicate points generally followed treatment- and stage-specific clustering patterns, while the greater dispersion observed in late-stage pathogen-stressed groups reflected increased biological heterogeneity of the rhizosphere metabolome under disease pressure. Importantly, the PCA included CK and *Bacillus*-only treatments across sampling stages, providing a baseline view of metabolic variation in the absence of pathogen pressure. Based on this baseline evaluation, subsequent differential metabolite, K-means clustering, and microbiome–metabolome association analyses focused on pathogen-stressed treatments, namely SR13, SBR13, NR13, and NBR13, to characterize soil-context-dependent Bacillus-associated metabolic responses under disease pressure. Globally, the rhizosphere metabolome was dominated by organooxygen compounds, carboxylic acids, fatty acyls, together with several secondary-metabolism-related classes, including prenol lipids, benzene and substituted derivatives, phenols, cinnamic acids and derivatives, flavonoids, and isoflavonoids (Figure 5B; Supplementary Figure S4), suggesting the coexistence of basal metabolic and defense-associated metabolic features.

At 13 dpi, heatmap and PLS-DA analyses showed clear metabolic separation among NR13, NBR13, SR13, and SBR13, with the PLS-DA model explaining 78.20% of total variance and showing strong model fit and predictive ability (R^2^Y = 0.998, Q^2^ = 0.988). NR13 represented a distinct pathogen-associated metabolic state under non-sterilized soil, whereas NBR13 formed an independent trajectory separated from NR13, indicating that under indigenous microbial backgrounds, *Bacillus* was associated with a distinct pathogen-response-related metabolic profile rather than merely attenuating pathogen effects (Supplementary Figures S5 and S6).

To evaluate whether these metabolic differences were more strongly associated with *Bacillus* intervention or soil microbial background, UpSet analysis was performed on pairwise differential metabolite overlaps (Figure 5C). *Bacillus*-associated comparisons (NR13 vs. NBR13; SR13 vs. SBR13) shared only 81 differential metabolites, with a low Jaccard similarity index of 0.059, indicating highly context-specific metabolic remodeling across soil backgrounds. In contrast, sterilization-associated comparisons (SR13 vs. NR13; SBR13 vs. NBR13) shared 207 metabolites and exhibited a substantially higher Jaccard index of 0.132, representing a 2.24-fold greater overlap than *Bacillus*-associated comparisons (χ^2^ = 44.03, *p* < 0.001). These results quantitatively suggested that *Bacillus*-associated metabolic shifts were highly soil-context-dependent, whereas sterilized versus non-sterilized soil differences were linked to broader and more conserved changes in core metabolic profiles. Thus, *Bacillus* was mainly associated with the direction of treatment-specific metabolic shifts, while the soil microbial background contributed to basal metabolic stability.

K-means clustering combined with KEGG enrichment further resolved these differential metabolites into functionally distinct dynamic modules (Figure 5D, Supplementary Figure S7). Cluster 1 represented a *Bacillus*-induced defense and metabolic recovery module enriched in D-amino acid metabolism, secondary metabolite biosynthesis, and antibiotic-related pathways, suggesting enhanced antimicrobial- and defense-associated metabolic potential, particularly under sterilized-soil conditions. Cluster 5 peaked in NBR13 and was enriched in cutin/suberine/wax biosynthesis, carotenoid metabolism, indole alkaloids, and polyketides, representing a *Bacillus*-associated metabolite cluster potentially linked to barrier-related metabolism and secondary defense-associated pathways under the non-sterilized background. In contrast, Cluster 4 peaked specifically in SR13 and was enriched in carotenoid, sesquiterpenoid/triterpenoid, steroid, and unsaturated fatty acid biosynthesis, indicating that under sterile-pathogen conditions, rhizosphere metabolism was associated with membrane remodeling, structural defense-related processes, and defensive secondary metabolism. Meanwhile, Cluster 3 remained relatively enriched under non-sterilized soil conditions but declined under sterilized soil conditions, with enrichment in nucleotide metabolism, lipid signaling, and basal metabolic pathways, suggesting that the non-sterilized microbial background contributed to maintaining basal metabolic homeostasis. Overall, *Bacillus* did not simply reverse pathogen-associated metabolic disruption but was associated with a shift from pathogen-driven stress profiles toward defense-oriented and homeostasis-supporting metabolic patterns, a process strongly influenced by soil microbial background.

### 2.5. Soil Microbial Background Shapes Bacillus-Associated Rhizosphere Microbiome–Metabolome Correlations Under Pathogen Stress

To evaluate how indigenous microbial communities were associated with *Bacillus* biocontrol functionality, integrated microbiome–metabolome analyses primarily focused on SR13 vs. SBR13 and NR13 vs. NBR13.

**Figure 6:**
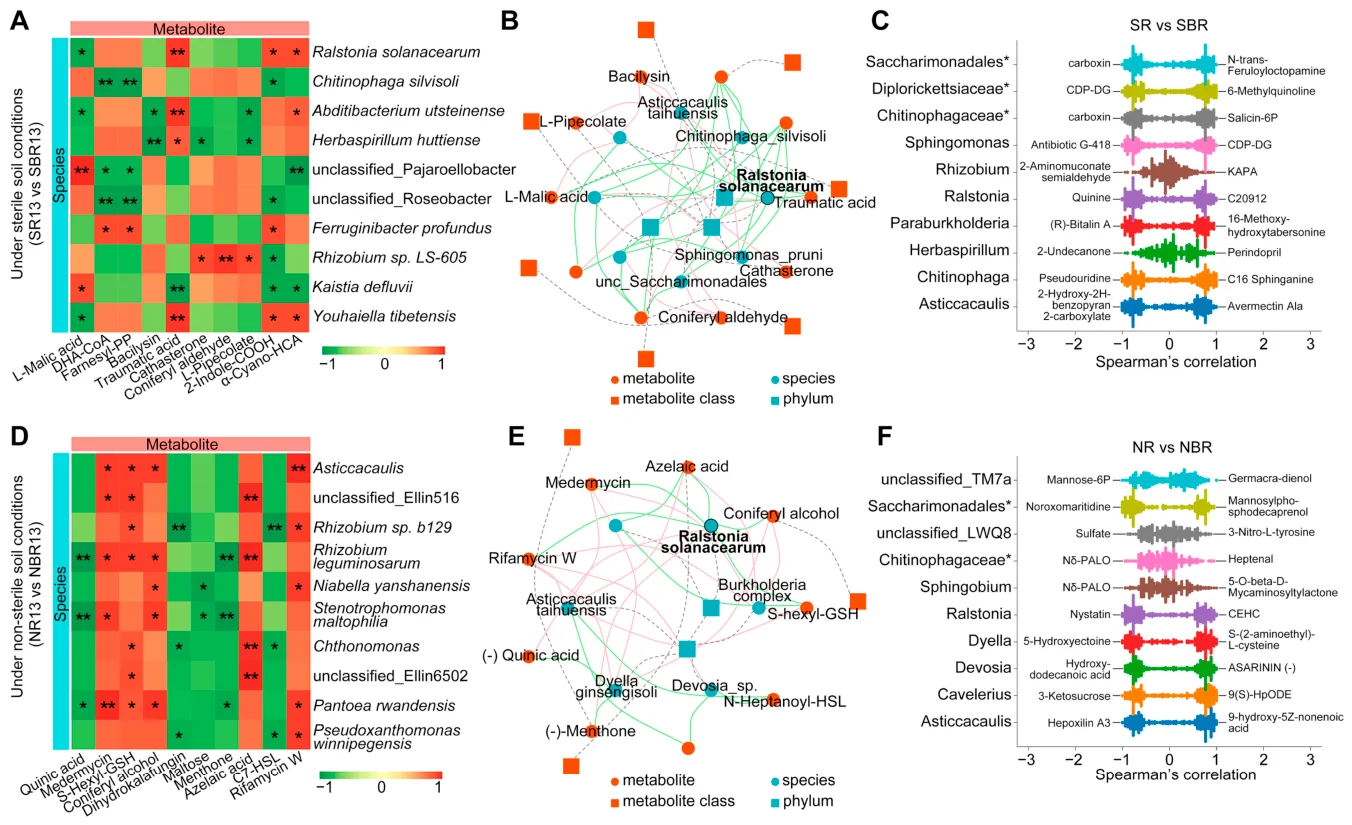
Integrated microbiome–metabolome correlation patterns under sterilized and non-sterilized soil conditions during *Bacillus*-associated pathogen suppression. (**A**) Heatmap showing correlations between major microbial taxa and differential metabolites under sterilized soil conditions (SR13 vs. SBR13). Asterisks indicate statistical significance (* *p* < 0.05; ** *p* < 0.01). (**B**) Integrated microbiome–metabolite correlation network under sterilized soil conditions. (**C**) Microbiota–metabolite axis analysis under sterilized soil conditions based on Spearman correlation distributions between representative taxa and key metabolites. For readability, unresolved taxa are shown without the “unclassified” prefix and marked with an asterisk (*). (**D**) Heatmap showing correlations between major microbial taxa and differential metabolites under non-sterilized soil conditions (NR13 vs. NBR13). Asterisks indicate statistical significance (* *p* < 0.05; ** *p* < 0.01). (**E**) Integrated microbiome–metabolite correlation network under non-sterilized soil conditions. (**F**) Microbiota–metabolite axis analysis under non-sterilized soil conditions based on Spearman correlation distributions between representative taxa and key metabolites. To improve readability, simplified node labeling was used in the main network panels (**B**,**E**), while the corresponding networks with complete node labels are provided in Supplementary Figure S8.

Under sterilized soil conditions, metabolite correlation analysis (Figure 6A) revealed two opposing microbiome–metabolite correlation patterns. *Ralstonia-*, *Abditibacterium-*, *Herbaspirillum-*, *Ferruginibacter-*, and *Youhaiella-associated ASVs* were broadly positively correlated with traumatic acid and metabolites putatively associated with membrane and lipid remodeling but negatively correlated with several metabolites, including L-malic acid, bacilysin, cathasterone, coniferyl aldehyde, and L-pipecolate, some of which have been previously associated with plant defense or antimicrobial functions. In contrast, taxa including *Chitinophaga*, *Rhizobium*, *Kaistia*, and *Pajaroellobacter* showed the opposite pattern, with stronger positive correlations with putative defense-related metabolites and negative correlations with metabolites potentially associated with pathogen-induced stress. Network analysis (Figure 6B) further showed that under sterilized soil conditions, microbiome–metabolite associations were dominated by convergent stress- and defense-related correlation patterns. Chitinophaga-, Saccharimonadales-, and Sphingomonas-related taxa were consistently negatively associated with damage- and membrane remodeling-related metabolites, whereas Asticcacaulis was more positively associated with structural defense-related metabolites. *Ralstonia* was mainly associated with pathogen damage-related metabolites but negatively correlated with L-malic acid, suggesting a close correlation between *Ralstonia* abundance and rhizosphere damage-associated metabolic profiles. Microbiota–metabolite axis analysis (Figure 6C) similarly showed that *Ralstonia* primarily tracked pathogen/damage-related metabolites, whereas *Chitinophaga*, *Asticcacaulis*, and *Herbaspirillum* were more strongly associated with membrane homeostasis, defense, and structural compensation-related metabolites.

In contrast, under non-sterilized soil conditions, metabolite–microbiome correlations showed an overall defense-oriented association structure (Figure 6D), where ASVs assigned to indigenous bacterial taxa displayed more differentiated metabolite association patterns. Major taxa, including *Asticcacaulis*, *Rhizobium*, *Niabella*, *Stenotrophomonas*, *Pantoea*, and *Pseudoxanthomonas*, were consistently positively associated with medermycin, S-hexyl glutathione, coniferyl alcohol, azelaic acid, and rifamycin W, while broadly negatively correlated with quinic acid, maltose, menthone, dihydrokalafungin, and N-heptanoylhomoserine lactone. These correlations suggested that Bacillus-associated rhizosphere metabolic patterns in non-sterilized soil were linked to metabolites previously implicated in aromatic metabolism, plant defense, and redox regulation, although their direct antimicrobial or resistance-inducing activities were not experimentally verified. Specifically, metabolite features putatively annotated as azelaic acid, coniferyl alcohol, and S-hexyl glutathione showed broad positive correlations with multiple indigenous bacterial ASVs. Features annotated as rifamycin W and medermycin, compounds with previously reported antimicrobial properties, were also associated with these bacterial ASVs. In contrast, the widespread negative associations with quinic acid, maltose, and the quorum-sensing-related metabolite N-heptanoylhomoserine lactone suggested reduced linkage with basal carbon flow and metabolites potentially involved in microbial signaling.

Network analysis (Figure 6E) further showed that, compared with the sterilized-soil network characterized by convergent compensatory responses, the non-sterilized network exhibited more differentiated microbiome–metabolite correlation patterns. Although *Ralstonia* remained a central node, *Ralstonia*-associated ASVs were negatively correlated with metabolite features putatively annotated as azelaic acid, coniferyl alcohol, and S-hexyl glutathione, suggesting that pathogen-associated microbial features were linked to distinct metabolic correlation patterns under non-sterilized soil conditions. Meanwhile, different indigenous bacterial taxa exhibited distinct microbiome–metabolome correlation patterns. *Burkholderia* was closely linked to putative defense-related metabolites, including azelaic acid, S-hexyl glutathione, rifamycin W, and coniferyl alcohol; *Devosia* was more associated with antimicrobial- and quorum-sensing-related metabolites; and *Dyella* was associated with both basal carbon flow- and aromatic defense-related metabolites. Microbiota–metabolite axis analysis (Figure 6F) similarly showed that *Dyella*, *Devosia*, and *Asticcacaulis* were more strongly associated with stress resistance-, aromatic defense-, and lipid homeostasis-related metabolites. Collectively, these results suggest that indigenous soil microbial background was associated with a shift from relatively uniform stress-related correlations toward more differentiated *Bacillus*-associated microbiome–metabolome correlation patterns under pathogen stress.

## 3. Discussion

### 3.1. Indigenous Microbiota Shapes the Functional Expression and Effectiveness of Bacillus Biocontrol

One of the central findings of this study is that *Bacillus* biocontrol efficacy was strongly associated with the soil microbial background. Although *Bacillus* partially reduced pathogen abundance and disease severity in sterilized soil, its suppressive effect was relatively limited, particularly during later disease progression. In contrast, under non-sterilized soil conditions, *Bacillus* treatment resulted in more sustained pathogen suppression, lower disease severity, higher microbial diversity, and distinct rhizosphere microbial and metabolic profiles. These findings suggest that the soil microbial background may influence the extent to which the biocontrol potential of *Bacillus* is expressed under pathogen stress. These associations do not establish direct functional cooperation between the introduced strain and indigenous microorganisms. Overall, these results support the hypothesis that differences in soil microbial background are associated with the efficacy and durability of *Bacillus*-mediated biocontrol, together with changes in pathogen abundance, rhizosphere microbial community structure, predicted functional potential, and defense-associated metabolic profiles.

These findings are consistent with broader ecological frameworks linking the core microbiome and classical disease-suppressive soil theory. Toju et al. [15] proposed that key indigenous taxa can serve as ecological scaffolds that amplify exogenous beneficial functions, while Finkel et al. [16] emphasized that successful establishment and persistence of introduced beneficial microbes depends on their integration into pre-existing microbiome networks. This aligns with the disease-suppressive soil framework [13], which suggests that pathogen suppression emerges from stable indigenous microbial networks rather than single antagonists. Subsequent studies further demonstrated that biocontrol effectiveness depends on resident microbiome interactions, ecological compatibility, and network integration [11,17,18]. Collectively, these studies support the view that biocontrol is not an isolated strain property but a community-context-dependent ecological process.

Building on this foundation, our study provides additional evidence that the performance of an introduced *Bacillus* strain can vary with the surrounding soil microbial background. In this context, differences in microbial enrichment, predicted functional potential, metabolite association patterns, and community-level associations may reflect distinct rhizosphere responses to *Bacillus* treatment under pathogen stress. As previous studies noted, beneficial microbial traits are highly dependent on compatibility with resident microbiomes, and microbial inoculant success depends less on strain identity alone than on network integration within pre-existing ecological systems [19,20]. Thus, variation in *Bacillus* performance across soils likely reflects differences in ecological context, particularly indigenous microbiota, pathogen pressure, and network compatibility, rather than strain inconsistency alone.

Therefore, indigenous microbiota should be viewed not merely as a background variable but as an ecological context that influences whether *Bacillus* remains a limited local suppressor or is associated with broader rhizosphere microbiome shifts, soil-context-dependent metabolic profiles, and defense-related microbial–metabolite patterns. However, the relative contributions of microbial community differences and sterilization-induced changes in soil physicochemical properties remain to be distinguished through further experimental validation.

### 3.2. Soil Microbial Background Shapes Distinct Bacillus-Associated Rhizosphere Response Patterns

Beyond influencing whether Bacillus can function effectively, our results further suggest that *Bacillus*-associated biocontrol did not follow a single fixed pattern but varied with the soil microbial background. This finding extends the conventional view of *Bacillus* primarily as a direct antagonist. *Bacillus* species have long been recognized for suppressing pathogens through antimicrobial lipopeptides, antibiotics, and volatile compounds [21], yet increasing evidence suggests that their ecological role in natural systems extends beyond direct antagonism to broader regulation of rhizosphere processes [19].

For example, a recent study demonstrated that fermentation filtrates and crude lipopeptide extracts from *Bacillus velezensis* TCS001 inhibited the growth, conidial germination, and virulence of *Magnaporthe oryzae*, while also activating defense-related responses in rice plants [22]. Although these findings highlight the diverse mechanisms of Bacillus-mediated biocontrol, the antifungal activities demonstrated in that system cannot be directly extrapolated to bacterial wilt suppression.

Under sterilized soil conditions in our study, where indigenous microbial buffering was largely removed and pathogen invasion rapidly shifted rhizosphere structure, *Bacillus* appeared to be associated mainly with partial disease buffering and localized stabilization. In this state, its effects were largely confined to delaying pathogen expansion, accompanied by changes in stress- and defense-associated microbial and metabolic profiles, resembling localized stabilization of a disturbed system rather than broad community-level recovery. This pattern is consistent with the resistance–resilience framework proposed by Shade et al. [23], in which external interventions in disturbed systems often first prevent further deterioration rather than immediately restore a fully coordinated ecological state.

In contrast, under non-sterilized soil conditions, *Bacillus* was associated with broader community and metabolic response patterns under pathogen pressure. *Bacillus* not only reduced the abundance-based dominance of *Ralstonia* but was also associated with a more connected but less centralized microbial association network, enrichment of potentially beneficial taxa including *Burkholderia*, *Devosia*, *Dyella*, and *Rhizobium*-related groups, and changes in predicted functional potential and metabolite association patterns. Previous studies emphasized that microbial competition is not solely resource competition but also involves reshaping ecological niches [24], and our results are consistent with this view under microbiome-intact conditions. Similarly, Kwak et al. showed in tomato bacterial wilt systems that disease resistance depends less on complete pathogen elimination than on rhizosphere restructuring into a more competitive ecological framework [25]. Consistent with this, the taxa enriched in our non-sterilized system were associated with defense-related metabolite profiles and predicted functional categories, suggesting potential links to aromatic compound metabolism and other ecological functions.

The full threshold-based networks further supported this soil-context-dependent interpretation. NBR showed a more connected but less centralized association pattern than NR, whereas SBR showed lower graph density and clustering coefficient than SR. Because *Ralstonia*-associated negative correlations did not uniformly increase across soil backgrounds, these network patterns should be interpreted as soil-dependent association shifts rather than direct evidence of antagonistic interactions. Thus, in the presence of indigenous microbiota, *Bacillus* treatment was associated with broader community-level and metabolic shifts under pathogen stress, beyond the observed reduction in pathogen abundance. These shifts may reflect partial buffering of pathogen-associated dysbiosis rather than direct evidence of active community redirection.

These contrasting response patterns may also be considered in the context of microbiome-assisted plant defense. Berendsen et al. (2012) proposed that plants can recruit beneficial microbes to strengthen immunity [12], while Carrión et al. (2019) further demonstrated that pathogen invasion can activate latent disease-suppressive functions within resident microbiomes [26]. Our findings extend this framework by suggesting that *Bacillus* may be associated with different rhizosphere response patterns depending on ecological context: toward damage-compensation-associated patterns in sterilized systems, or defense-related microbial and metabolite association patterns in non-sterilized systems. Thus, the same Bacillus strain may be associated with different microbial and metabolic responses depending on the soil environment. As Trivedi et al. emphasized, the future of plant microbiome research lies not only in identifying microbial members but also in understanding which organisms shape system function [20]. Our study further suggests that the value of *Bacillus* may lie not only in its isolated antimicrobial capacity but also in its potential association with soil-context-dependent rhizosphere responses under pathogen stress.

### 3.3. Implications for Soil-Dependent Biocontrol Performance

One of the most direct practical implications of this study is that it provides a possible ecological explanation for variation in *Bacillus*-mediated disease suppression under different soil conditions. Traditional biocontrol strategies have largely operated under a strain-centered framework, assuming that once a microbial strain with strong in vitro antagonism, plant growth promotion, or induced resistance potential is identified, it can function broadly as a universal inoculant. This model essentially treats biocontrol as a strain property. However, both field inconsistency and recent microbiome research increasingly suggest that this view oversimplifies the ecological complexity of natural soils. Previous studies argued that one major reason microbial inoculants frequently fail to translate from controlled systems to field performance is the ecological resistance imposed by resident microbiomes [27]. Similarly, Kaminsky et al. emphasized that microbial inoculant success depends strongly on interactions with indigenous community structure, resource competition, and pathogen pressure, rather than on strain traits alone [28].

Our results provide experimental evidence that the same *B. subtilis* treatment exhibited different levels of bacterial wilt suppression under contrasting soil backgrounds. Notably, stronger disease suppression in non-sterilized soil occurred despite lower detected *gyrB* gene abundance than in sterilized soil, suggesting that inoculant abundance alone could not fully explain the observed differences in biocontrol performance. The accompanying differences in microbial community composition, predicted functional potential, and metabolite association patterns further suggest that the soil microbial background may contribute to these contrasting responses. Previous studies demonstrated that initial soil microbiome composition can strongly predict future plant health [11,29], while Trivedi et al. further argued that plant health management should shift from single-strain inoculation toward microbiome assembly control [20]. Together, these findings highlight the importance of considering soil microbial background when evaluating the performance of *Bacillus*-based biocontrol agents. Nevertheless, these findings were obtained under controlled pot conditions and do not establish the effectiveness of soil-adaptive deployment strategies under field conditions. Future microbial reconstitution and field studies across different soil types are needed to determine the broader applicability of these observations.

### 3.4. Limitations and Future Perspectives

Several limitations should be considered. First, although autoclaving simplified the soil microbial background, it may also have altered nutrient availability, soluble organic compounds, pH, and soil structure, potentially influencing pathogen growth, plant responses, and *Bacillus* performance [30,31]. Thus, differences between sterilized and non-sterilized soils cannot be attributed solely to indigenous microbiota depletion. Future studies incorporating soil physicochemical measurements, gamma irradiation, or microbial reconstitution could help distinguish microbial from sterilization-induced abiotic effects. Furthermore, despite aseptic handling, sterile irrigation, bottom watering, and physical separation, greenhouse conditions could not completely exclude microbial recolonization of sterilized soil. Although *gyrB* primer specificity was evaluated using NCBI Primer-BLAST online tool (National Center for Biotechnology Information, Bethesda, MD, USA). and non-inoculated controls were included, potential cross-amplification from indigenous *Bacillus* species also warrants further experimental validation.

Second, although CK and *Bacillus*-only treatments were included in the overall sequencing and metabolomic ordination analyses, detailed co-occurrence, K-means clustering, and microbiome–metabolome analyses focused on pathogen-stressed treatments. Therefore, the observed patterns primarily reflect *Bacillus*-associated responses under pathogen pressure rather than its general effects in the absence of disease. Moreover, correlation-based networks cannot establish causality or functional synergy between *B. subtilis* and indigenous bacterial taxa, including *Burkholderia* and *Devosia*. Similarly, the proposed antimicrobial and defense-related functions of selected metabolites were inferred from untargeted metabolomics and previous literature without absolute quantification or direct validation of antimicrobial and ISR-inducing activities. Targeted metabolite quantification, co-culture assays, synthetic community experiments, and plant inoculation assays are therefore needed to validate the roles of key taxa and metabolites, including azelaic acid, coniferyl alcohol, rifamycin W, and medermycin.

Finally, this controlled pot experiment included three independent pot-level biological replicates per treatment, potentially limiting statistical power for variable microbiome and metabolome data. Future studies with larger sample sizes (ideally ≥5–6 independent biological replicates) and field trials across diverse soil types and ecological backgrounds are needed to assess the broader applicability of soil-dependent *Bacillus* biocontrol strategies.

## 4. Materials and Methods

### 4.1. Experimental Design and Soil Preparation

A pot-based controlled experiment was conducted to evaluate the effects of soil microbial background on potato bacterial wilt development and the biocontrol efficacy of *B. subtilis*. Non-sterilized soil used in this study was collected in May 2025 from the topsoil layer (0–20 cm) of a potato field located in Butuo County, Liangshan Prefecture, Sichuan Province, China, with no recent history of severe soil-borne disease. After removing plant residues and stones, the soil was thoroughly homogenized to preserve its natural indigenous microbial community structure. A portion of the same homogenized soil was sterilized by autoclaving at 121 °C twice at 24 h intervals to generate sterilized soil with a simplified microbial background. The effectiveness of soil sterilization was checked by plating soil suspensions from sterilized soil onto nutrient agar plates before transplantation and inoculation, and no visible colony growth was observed after incubation. Because autoclaving may also alter soil physicochemical properties, including nutrient availability, soluble organic compounds, pH, and soil structure, the sterilized soil treatment was interpreted as a combined perturbation involving both indigenous microbiota depletion and potential sterilization-induced abiotic changes. After sterilization, the sterilized soil was cooled, mixed, and transferred into sterilized pots under clean-bench conditions using sterilized trays, tools, and gloves. The soil surface was covered with sterilized gauze after potting to reduce airborne microbial deposition while maintaining gas exchange.

Uniform tissue-cultured potato plantlets were used. Transplantation into sterilized soil was performed under clean conditions using sterilized tools. Plants in sterilized soil were placed in sterilized trays and physically separated from non-sterilized soil treatments to reduce cross-contamination. Irrigation was performed with sterile water or sterile nutrient solution using bottom watering. Following transplantation, all plants were maintained under open greenhouse conditions rather than gnotobiotic conditions, with a temperature of 24 °C, relative humidity of 60%, and a 16 h light/8 h dark photoperiod. Although multiple aseptic handling and separation procedures were applied, recolonization of sterilized soil by airborne microorganisms or microorganisms from the surrounding greenhouse environment could not be completely excluded.

The experiment followed a factorial design consisting of soil background and inoculation treatment. Soil background included non-sterilized soil (N) and sterilized soil (S). For rhizosphere microbiome, pathogen quantification, and metabolomic analyses, three inoculation treatments were included: *Bacillus subtilis* inoculation (B), *Ralstonia solanacearum* inoculation (R), and combined *Bacillus*+*Ralstonia* inoculation (BR). Thus, six treatment combinations were analyzed: NB, NR, NBR, SB, SR, and SBR. Each treatment combination consisted of three independent biological replicates, with each replicate represented by one pot containing three potato plants. Therefore, the pot was considered the experimental unit for statistical analysis, and plant-level measurements within each pot were averaged at the pot level before analysis.

### 4.2. Bacterial Strains, Inoculum Preparation, and Sample Collection

The potato cultivar used in this study was ‘Favorita’. The *B. subtilis* and *R. solanacearum* strains used in this study were isolated from a potato bacterial wilt field in Butuo County, Liangshan Prefecture, Sichuan Province, China, and were preserved and provided by Xichang University. Both strains were activated in nutrient agar (NA) liquid medium. Single colonies of *B. subtilis* and *R. solanacearum* were separately inoculated into 5 mL NA liquid medium and cultured at 30 °C and 180 r/min for 24 h as seed cultures. The seed cultures were then transferred at a 1‰ inoculation ratio into 2 L Erlenmeyer flasks containing 500 mL NA liquid medium and cultured at 30 °C and 180 r/min for another 24 h. After cultivation, bacterial suspensions were centrifuged at 4 °C and 4000 r/min for 10 min. The supernatant was discarded, and bacterial pellets were washed twice with sterile water, resuspended, and adjusted to 1 × 10^8^ CFU/mL for inoculation.

For inoculation, roots were mechanically wounded prior to rhizosphere drenching. In the B and R treatment, each plant received 10 mL of *B. subtilis* suspension and 10 mL of *R. solanacearum* suspension, respectively. In the BR treatment, each plant was first inoculated with 10 mL of *B. subtilis* suspension, followed by 10 mL of *R. solanacearum* suspension 24 h later. Both inocula were applied to the root zone by rhizosphere drenching after root wounding. Mock-treated plants received an equal volume of sterile water following root wounding and were used as non-inoculated controls where applicable. Inoculation volume and concentration were kept consistent across all corresponding treatments.

To monitor temporal changes in disease progression and rhizosphere responses, samples were collected at 0 days post inoculation (dpi, before pathogen inoculation), 6 dpi (early stage), and 13 dpi (late stage). At each sampling point, three independent pots were sampled for each soil background × treatment combination. Accordingly, a total of 54 rhizosphere samples were collected for downstream analyses, corresponding to 2 soil backgrounds × 3 inoculation treatments × 3 biological replicates × 3 sampling time points. For each biological replicate, rhizosphere soil from the three plants within the same pot was pooled to generate one composite pot-level sample. Plants were carefully uprooted, loosely attached soil was gently removed, and rhizosphere soil tightly adhering to root surfaces was collected. All rhizosphere samples were processed under sterile conditions, immediately frozen in liquid nitrogen, and stored at −80 °C for subsequent full-length 16S rRNA sequencing, *R. solanacearum* quantification, and LC-QTOF-based untargeted metabolomic analyses.

### 4.3. Disease Index Assessment

Disease severity was evaluated according to the method of Timila and Manandhar (2016) based on visible wilting symptoms [32]. Plants showing leaf wilting were considered diseased [33]. Disease severity was scored on a 0–4 scale as follows: 0, no wilting; 1, 1–25% of leaves wilted; 2, 26–50% of leaves wilted; 3, 51–75% of leaves wilted; and 4, 76–100% of leaves wilted. Disease index (DI) was calculated according to Xue et al. [34] using the formula: DI (%) = [Σ (number of plants at each disease grade × corresponding grade)/(total number of plants × highest grade)] × 100.

### 4.4. Quantification of R. solanacearum and B. subtilis Abundance by qPCR

To quantify *R. solanacearum* abundance in rhizosphere soil, quantitative real-time PCR (qPCR) targeting the *fliC* gene was performed using the primer pair fliC-F (5′-GAACGCCAACGGTGCGAACT-3′) and fliC-R (5′-GGCGGCCTTCAGGGAGGTC-3′). The *fliC* primer pair was selected based on previous studies and further checked for specificity using NCBI Primer-BLAST. Standard curves were generated using 10-fold serial dilutions of purified plasmid DNA containing the target *fliC* gene fragment with known copy numbers, and Ct values were converted to gene copy numbers based on the resulting standard curve. Pathogen abundance in each sample was then normalized according to the initial soil weight used for DNA extraction and expressed as copies/g soil.

To evaluate the abundance of the introduced *B. subtilis* in the rhizosphere, qPCR targeting the *B. subtilis gyrB* gene was further performed using DNA extracted from the same rhizosphere soil samples. Mock and *Ralstonia*-only samples were included as non-*Bacillus*-treated controls to evaluate background amplification. The primer pair gyrB_5234-F (5′-CGGTCGTAAACGCACTATC-3′) and gyrB_5391-R (5′-AGGGTCCGGGACAAAATGTGTCG-3′) were adapted from Xie et al. [35] Primer specificity was further checked using NCBI Primer-BLAST. The identity of the target amplicon was further verified by conventional PCR followed by Sanger sequencing using genomic DNA from the pure-cultured *B. subtilis* strain. Standard curves were generated using 10-fold serial dilutions of purified plasmid DNA containing the target *gyrB* fragment with known copy numbers, and Ct values were converted to *gyrB* gene copy numbers based on the standard curve. *B. subtilis* abundance was normalized according to the initial soil weight used for DNA extraction and expressed as copies/g soil. All samples were analyzed with technical replicates to ensure quantification accuracy.

### 4.5. DNA Extraction, Full-Length 16S rRNA Amplification, and PacBio Sequencing

Total genomic DNA from rhizosphere soil samples was extracted using the TGuide S96 Magnetic Soil/Stool DNA Kit (Tiangen Biotech, Beijing, China) according to the manufacturer’s instructions. DNA quality and concentration were assessed by 1.8% agarose gel electrophoresis and a NanoDrop 2000 UV–Vis spectrophotometer (Thermo Fisher Scientific, Wilmington, DE, USA).

For bacterial community analysis, full-length 16S rRNA genes were amplified using barcoded universal primer pairs 27F (5′-AGRGTTTGATYNTGGCTCAG-3′) and 1492R (5′-TASGGHTACCTTGTTASGACTT-3′). PCR amplification was performed using KOD One PCR Master Mix (TOYOBO Life Science, Toyobo Co., Ltd., Osaka, Japan) under the following conditions: initial denaturation at 95 °C for 2 min; 25 cycles of 98 °C for 10 s, 55 °C for 30 s, and 72 °C for 1 min 30 s; followed by a final extension at 72 °C for 2 min.

PCR products were purified using VAHTS DNA Clean Beads (Vazyme, Nanjing, China) and quantified using the Qubit dsDNA HS Assay Kit with a Qubit 3.0 Fluorometer (Invitrogen, Thermo Fisher Scientific, Carlsbad, CA, USA). Equimolar amplicons from all samples were pooled, and SMRTbell libraries were constructed using the SMRTbell Express Template Prep Kit 2.0 (Pacific Biosciences, Pacific Biosciences of California, Inc., Menlo Park, CA, USA). Library sequencing was performed on the PacBio Sequel II platform (Beijing Biomarker Technologies, Beijing, China) for single-molecule real-time (SMRT) sequencing. Full-length 16S rRNA sequencing data were used for downstream analyses of rhizosphere bacterial community composition and diversity across treatments.

### 4.6. Sequence Processing, Taxonomic Annotation, and Microbial Diversity Analysis

Raw circular consensus sequencing (CCS) reads generated from the PacBio Sequel II platform were quality-filtered and denoised using the DADA2 pipeline implemented in QIIME2 to generate high-resolution amplicon sequence variants (ASVs) [36]. Low-abundance ASVs with extremely low total counts across all samples were removed to minimize potential noise. Representative ASVs were taxonomically classified using the Naive Bayes classifier within QIIME2 against the SILVA ribosomal RNA database (release 138.1) with a confidence threshold of 70% [37,38].

To evaluate within-sample microbial diversity, alpha diversity indices including Shannon and Chao1 indices were calculated in QIIME2. Between-sample community dissimilarity (beta diversity) was assessed based on Bray–Curtis distance matrices and visualized using principal coordinate analysis (PCoA) in R (version 4.2.2) with the vegan package. Hierarchical clustering based on UPGMA was performed to compare overall community relationships among samples [39]. GraPhlAn (Graphical Phylogenetic Analysis) was used to construct circular cladograms based on taxonomic annotation results [40]. Statistical testing of the main effects and interactions among experimental factors on diversity was described in the Statistical analysis section.

### 4.7. Differential Taxonomic Analysis, Biomarker Identification, and Functional Prediction

Differential abundance analysis was performed using Linear Discriminant Analysis Effect Size (LEfSe, v1.1.1) [41] through the Galaxy platform. Taxa with low overall abundance were filtered prior to analysis to reduce noise, and taxonomic features with logarithmic LDA scores above the selected threshold were considered key discriminatory biomarkers. Random Forest (RF) classification analysis was conducted in R using the randomForest package [42]. Taxonomic abundance tables at multiple hierarchical levels were used as input variables. Model performance was evaluated using cross-validation, and taxa with the highest importance values were defined as major biomarkers contributing to group differentiation. Predicted functional potential of bacterial communities were predicted using the FAPROTAX database [43], which maps taxonomic identities to putative functional categories. Pairwise comparisons were performed using the Wilcoxon rank-sum test where appropriate, and *p* values were adjusted using the false discovery rate method. FAPROTAX annotations were interpreted as literature-based predictions of potential ecological functions rather than direct measurements of functional gene abundance, gene expression, or metabolic activity.

### 4.8. Microbial Co-Occurrence Network Analysis

Microbial co-occurrence networks were constructed for the NR, NBR, SR, and SBR groups at 13 dpi based on genus-level bacterial relative abundance profiles. Pairwise associations among bacterial genera were evaluated using Spearman’s rank correlation analysis, and correlation *p* values were adjusted using the Benjamini–Hochberg false discovery rate procedure. For each treatment group, full threshold-based networks were constructed by retaining all significant associations with FDR-adjusted *p* < 0.05 and |ρ| > 0.8. Positive and negative coefficients were represented as positive and negative associations, respectively.

Network topological properties, including node number, edge number, average degree, average path length, graph diameter, graph density, clustering coefficient, centralization, and modularity, were calculated from the full threshold-based networks. Ralstonia-associated features, including Ralstonia degree, degree centrality, and the numbers of positive and negative edges directly connected to Ralstonia, were also calculated from the full networks. To improve readability in the main figure, simplified networks containing the top 30 most abundant genera from each full network were visualized using Gephi (version 0.10.1), with all threshold-qualified edges among these selected genera retained. Each node represents a bacterial genus, node size represents its relative abundance, and edge color indicates the direction of the correlation. The simplified networks were used only for visualization, whereas all quantitative network comparisons were based on the full threshold-based networks.

### 4.9. Metabolite Extraction and LC-QTOF-MS Analysis

Rhizosphere soil metabolites were extracted using a modified methanol/acetonitrile/water protocol. Briefly, 50 mg of frozen rhizosphere soil was mixed with extraction solvent (methanol:acetonitrile:water, 2:2:1, *v*/*v*/*v*) containing L-2-chlorophenylalanine as an internal standard. Samples were vortexed, homogenized with ceramic beads, and subjected to ultrasonic extraction under ice-bath conditions to enhance metabolite release. After incubation at −20 °C to promote protein precipitation, samples were centrifuged at 4 °C, and the supernatant was collected. The extract was subsequently diluted with LC-MS grade water, filtered through a 0.22 μm membrane, and transferred to sample vials for metabolomic analysis.

Untargeted metabolomic profiling was performed using an ultra-performance liquid chromatography system Waters UPLC I-Class PLUS (Waters Corporation, Milford, MA, USA) coupled with a Xevo G2-XS QTOF high-resolution mass spectrometer (Waters, USA). Chromatographic separation was conducted on a Waters Acquity UPLC HSS T3 column (2.1 mm × 100 mm, 1.8 μm). Mobile phases consisted of water with 0.1% formic acid (phase A) and acetonitrile with 0.1% formic acid (phase B), and samples were analyzed under both positive and negative electrospray ionization modes using gradient elution.

Mass spectrometry data were acquired in MSE mode to simultaneously obtain precursor ion and fragmentation information. Source parameters, including capillary voltage, cone voltage, ion source temperature, and desolvation conditions, were optimized according to instrument standards to ensure stable signal acquisition. Six quality control (QC) samples were included to assess the analytical stability and reproducibility of the LC-QTOF-MS platform. QC performance was evaluated using total ion chromatograms (TICs), PCA clustering, pairwise Spearman correlations, and the relative standard deviation (RSD) of peak intensities across QC samples.

### 4.10. Metabolomic Data Processing, Annotation, and Pathway Analysis

Raw LC-MS data were processed using Progenesis QI v2.3 (Nonlinear Dynamics, Newcastle, UK) for peak detection, retention time alignment, and feature extraction. Metabolic features with missing values in more than 50% of samples were removed and remaining zero values were replaced with half of the minimum detected intensity.

Metabolite annotation was performed by matching accurate mass spectra, MS/MS fragmentation patterns, and isotope distribution information against the METLIN database [44] and an in-house reference library (Biomarker Biotech, Beijing, China). The matching parameters included a precursor ion tolerance of 5 ppm, a product ion tolerance of 10 ppm, and a product ion intensity threshold of 5%. Only annotations with matching scores ≥ 36 out of 60 were retained. According to the Metabolomics Standards Initiative (MSI) framework, compounds supported by reliable MS/MS spectral library matching without confirmation using authentic reference standards were considered putatively annotated metabolites (Level 2). Annotations lacking sufficient structure-specific MS/MS evidence were regarded as tentative and were not considered confidently identified compounds.

Principal component analysis (PCA) and partial least squares discriminant analysis (PLS-DA) were performed to evaluate overall metabolic variation, sample separation, and model performance [45]. Functional annotation of identified metabolites was conducted using the Kyoto Encyclopedia of Genes and Genomes (KEGG) database [46], and pathway enrichment analyses were performed using MetaboAnalyst v5.0 [47] to identify significantly altered metabolic pathways associated with soil microbial background, pathogen stress, and *Bacillus* intervention. Spearman correlation analyses were used to evaluate metabolite–microbiome association patterns and support integrated microbiome–metabolome network analyses.

### 4.11. Statistical Analysis

All statistical analyses were performed using pot-level biological replicates, with each pot considered as one independent experimental unit. For each pot, measurements from the three plants were averaged before statistical analysis. Because independent pots were sampled at each time point, sampling time was treated as a fixed factor rather than a repeated-measures factor.

For disease index and *R. solanacearum* abundance, only pathogen-inoculated treatments were included. At each time point, two-way ANOVA was used to test the effects of soil background, *Bacillus* treatment, and their interaction. For *B. subtilis* abundance, *Bacillus*-treated groups were included, and data at each time point were analyzed using two-way ANOVA with soil background, pathogen presence, and their interaction as fixed effects. qPCR data were log_10_-transformed before statistical analysis to improve normality and homogeneity of variance. When significant main effects or interactions were detected, multiple comparisons among treatment groups were performed using Tukey’s HSD test.

For microbial alpha diversity indices, factorial analysis was used to test the main effects and interactions of soil background, inoculation treatment, and sampling time. When assumptions of normality or homogeneity of variance were not met, Kruskal–Wallis tests followed by pairwise Wilcoxon rank-sum tests were used for group-wise comparisons. Beta diversity differences based on Bray–Curtis distances were evaluated using PERMANOVA with 999 permutations, with soil background, inoculation treatment, sampling time, and their interaction terms included in the model. ANOSIM was additionally used to assess group separation.

For differential taxonomic and predicted functional analyses, Kruskal–Wallis tests followed by pairwise Wilcoxon rank-sum tests were used where appropriate, and *p* values were adjusted using the false discovery rate method. For metabolomic data, differential metabolites were identified based on VIP > 1, *p* < 0.05, and fold-change thresholds in predefined pairwise comparisons. Spearman correlation analyses were used to evaluate microbiome–metabolome associations, and correlation *p* values were adjusted using the Benjamini–Hochberg false discovery rate procedure where applicable. Unless otherwise stated, statistical significance was defined at *p* < 0.05.

## 5. Conclusions

**Figure 7:**
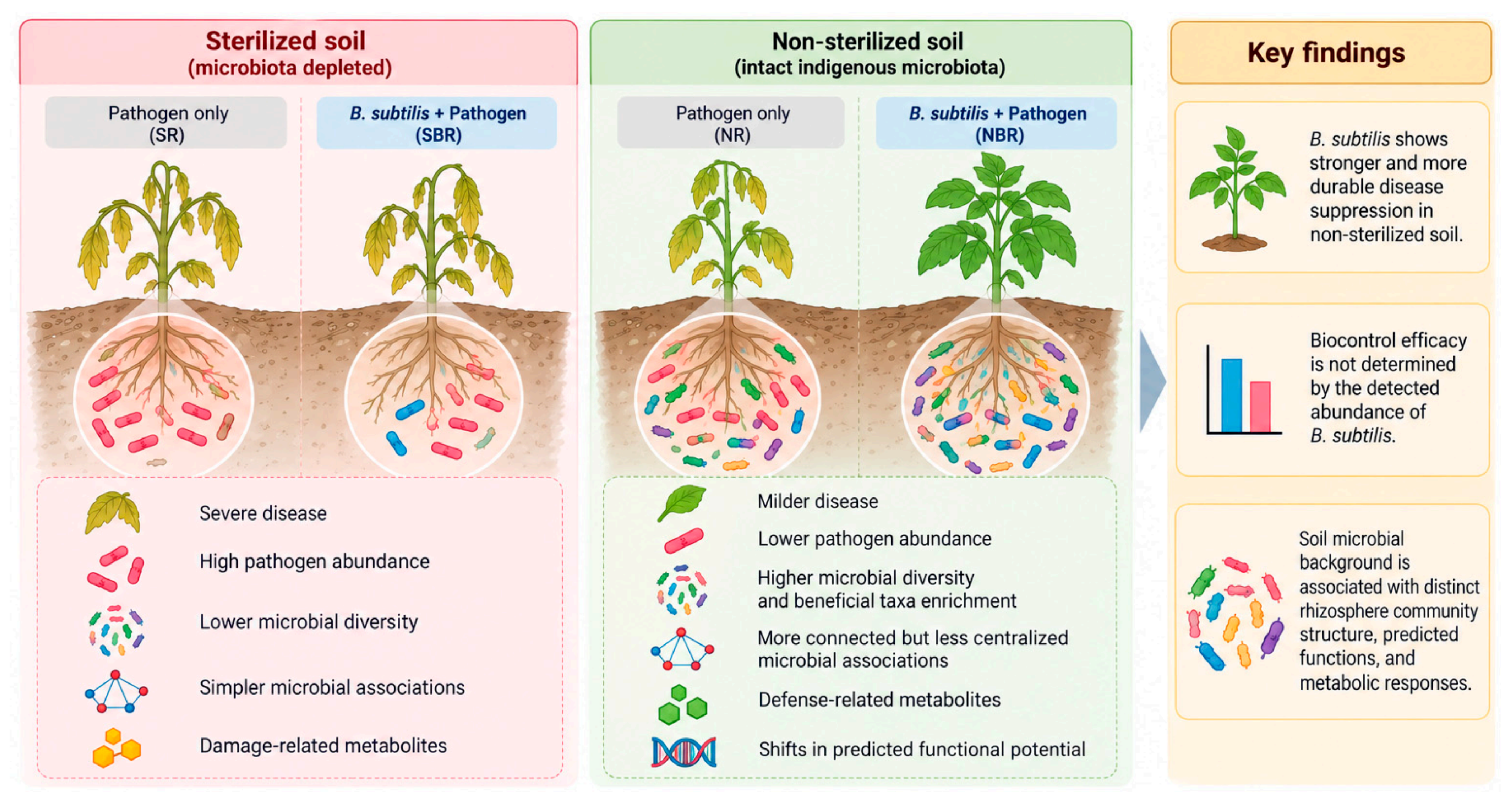
Schematic overview of soil-background-dependent *B. subtilis*-mediated biocontrol against potato bacterial wilt. Under sterilized soil conditions, *B. subtilis* treatment was associated with limited pathogen suppression, reduced microbial diversity, and predominantly stress-associated metabolic profiles. In contrast, non-sterilized soil exhibited stronger and more sustained disease suppression following *B. subtilis* treatment, accompanied by greater microbial diversity, distinct microbial association networks, shifts in predicted functional potential, and defense-associated metabolic profiles.

This study demonstrated that soil background was strongly associated with differences in the efficacy of *Bacillus subtilis*-mediated biocontrol against potato bacterial wilt. Although *B. subtilis* was detected at substantial levels in both sterilized and non-sterilized rhizosphere soils, stronger and more durable suppression of *R. solanacearum* occurred under the non-sterilized soil background. This suggested that differences in biocontrol efficacy could not be explained solely by the detected abundance of the introduced *Bacillus* but were also associated with the contrasting soil microbial backgrounds. Integrated microbiome and metabolome analyses further showed that the non-sterilized microbial background was associated with reduced pathogen dominance, enrichment of potentially beneficial relevant taxa, soil-dependent microbial association patterns, shifts in predicted functional potential, and distinct defense-associated metabolic profiles. In contrast, sterilized soil showed limited community buffering, stronger pathogen rebound, and more damage-compensation-associated metabolic patterns. These findings suggested that soil microbial background may influence the observed effectiveness of *Bacillus*-mediated biocontrol, accompanied by distinct rhizosphere community shifts and microbiome–metabolome association patterns under pathogen stress. The major findings and soil-dependent rhizosphere response patterns are summarized schematically in Figure 7. Overall, this study highlighted the importance of considering soil microbial background when evaluating *Bacillus*-mediated biocontrol against potato bacterial wilt, while further functional validation and field studies are needed to establish its practical implications.

## Data Availability

The data presented in the study are deposited in the National Center for Biotechnology Information (NCBI) Sequence Read Archive (SRA) database, BioProject accession number PRJNA1468432.

## Supplementary Materials

The following supporting information can be downloaded at: https://www.mdpi.com/article/10.3390/plants15213252/s1, Figure S1: Species-level taxonomic composition of rhizosphere bacterial communities across soil backgrounds, treatments, and disease progression stages. Relative abundance bar plots showing dominant bacterial taxa at the species level across sterilized (S) and non-sterilized (N) soil systems under Mock, *Bacillus* (B), *Ralstonia* (R), and *Bacillus*+*Ralstonia* (BR) treatments at 0, 6, and 13 dpi. Major taxa are displayed individually, while low-abundance taxa are grouped as Others; Figure S2: GraPhlAn phylogenetic comparisons of *Bacillus*-mediated rhizosphere community shifts under pathogen stress in sterilized and non-sterilized soils. (A) GraPhlAn phylogenetic cladogram comparing SR13 and SBR13 communities under sterilized soil conditions. (B) GraPhlAn phylogenetic cladogram comparing NR13 and NBR13 communities under non-sterilized soil conditions; Figure S3: Quality control (QC) assessment of the LC-QTOF-MS metabolomics data. (A), PCA score plot including all biological samples and six QC samples. (B), Spearman correlation heatmap of QC samples. (C), Distribution of relative standard deviation (RSD) values for peak intensities across QC samples. (D), Representative total ion chromatograms (TICs) of QC samples in negative ionization mode (ESI−). Figure S4: KEGG pathway classification of annotated rhizosphere metabolites detected by untargeted LC-QTOF metabolomics; Figure S5: Hierarchical clustering heatmap of differential metabolite abundance among NR13, NBR13, SR13, and SBR13 groups. Heatmap showing normalized abundance patterns (Z-score) of significantly differential metabolites across four major treatment groups at 13 dpi. Rows represent metabolites, columns represent biological replicates, and hierarchical clustering illustrates treatment-associated metabolic differentiation; Figure S6: PLS-DA and permutation validation of rhizosphere metabolomic differentiation among NR13, NBR13, SR13, and SBR13 groups at 13 dpi. (A) Partial least squares discriminant analysis (PLS-DA) score plot showing separation of metabolomic profiles among treatment groups. (B) Permutation test validating the robustness and predictive reliability of the PLS-DA model based on R^2^Y and Q^2^Y values; Figure S7: KEGG pathway enrichment analysis of K-means-derived differential metabolite clusters. The *x*-axis represents rich factor, bubble size indicates fold enrichment, and bubble color represents enrichment significance (*p* value). Each panel corresponds to one dynamic metabolite cluster and its dominant functional pathway categories; Figure S8: Microbiome–metabolome correlation networks with complete node labels under sterilized and non-sterilized soil conditions. (A) SR13 vs. SBR13; (B) NR13 vs. NBR13. The networks correspond to Figure 6B and 6E, respectively, with complete node labels provided for reference; Table S1: Topological properties of full threshold-based rhizosphere bacterial co-occurrence networks at 13 dpi.
